# Preparation and Flame Retardance of Polyurethane Composites Containing Microencapsulated Melamine Polyphosphate

**DOI:** 10.3390/polym9090407

**Published:** 2017-08-31

**Authors:** Shang-Hao Liu, Chen-Feng Kuan, Hsu-Chiang Kuan, Ming-Yuan Shen, Jia-Ming Yang, Chin-Lung Chiang

**Affiliations:** 1 Department of Ammunition Engineering and Explosion Technology, Anhui University of Science and Technology, Huainan 232001, China; shliu998@163.com; 2 Department of Food and Beverage Management, Far East University, Tainan City 74448, Taiwan; cfkuan@mail.feu.edu.tw (C.-F.K.); hckuan@mail.feu.edu.tw (H.-C.K.); 3 Department of Chemical and Materials Engineering, Southern Taiwan University of Science and Technology, Tainan City 71005, Taiwan; hbj678@gmail.com; 4 Green Flame Retardant Material Research Laboratory, Department of Safety, Health and Environmental Engineering, Hung-Kuang University, Taichung City 43302, Taiwan; young1362@gmail.com

**Keywords:** microencapsulation, melamine polyphosphate, polyurethane, composite, flame retardant

## Abstract

A new microencapsulated flame retardant containing melamine polyphosphate (MPP) and 4,4′-oxydianiline-formaldehyde (OF) resin as the core and shell materials, respectively, was synthesized by in situ polymerization. ^29^Si NMR was used to measure the condensation density of polyurethane containing silicon compound (Si-PU). The structures and properties of the microencapsulated melamine polyphosphate (OFMPP) were characterized using X-ray photoelectron spectroscopy, scanning electron microscopy and water solubility. Thermal behavior of the OFMPP was systematically analyzed through thermogravimetric analysis. Flame retardance tests such as the limiting oxygen index and UL-94 were employed to evaluate the effect of composition variation on the MPP and OFMPP in polyurethane composites. The results indicated that the microencapsulation of MPP with the OF resin improved hydrophobicity and that the flame retardance of the Si-PU/OFMPP composite (limiting oxygen index, LOI = 32%) was higher than that of the Si-PU/MPP composite (LOI = 27%) at the same additive loading (30 wt %).

## 1. Introduction

Polyurethane (PU) can be manufactured as per different requirements and is widely applied to fulfill daily needs and manufacture industrial goods. PU can be applied to coatings, adhesives, synthetic leather and elastomers [1,2]. Although its applications are broad, its thermal stability and flame retardance are relatively poor owing to its structure, limiting its application in certain areas. In recent years, many studies have been conducted on flame-retardant PU and considerably high flame retardance has been achieved [1,2,3]. In this study, silicon component was introduced into PU to increase thermal stability of polymer matrix, which was called as Si-PU. It was expected to expand above applications fields. Recently, commercial halogen-free flame retardants have gained increasing research attention. Several series of phosphorus and nitrogen compounds such as phosphates [4], melamine [5], ammonium polyphosphate (APP) [6], melamine polyphosphate (MPP) [7] and melamine phosphate (MP) [8] have been studied. The present study focused on MPP. Because MPP has two types of structures, APP and melamine, it can promote the formation of a char layer at the surface of the polymer via phosphorylation and promotion of cationic crosslinking during the flame retardation process, achieving an intumescent effect [9]. The effect of an intumescent flame retardant is thus related to char formation; however, char formation is also related to the level of intumescent flame retardant present and thermal degradation properties [5]. In recent years, microencapsulated flame retardants have been widely studied by scholars who believe that MPP in microcapsules can effectively improve flame retardance and thermal stability [10,11,12,13,14,15]. In this study, microencapsulation of MPP was utilized to obtain a surface with a char-forming compound. Furthermore, microencapsulated MPP (4,4′-oxydianiline-formaldehyde MPP (OFMPP)) was prepared and used. The thermal stability and flame retardance of the composites containing microencapsulated MPP were determined.

## 2. Materials and Methods 

### 2.1. Materials

Isophorone diisocyanate (IPDI, purity 98%), ethylenediamine (purity 99%), 3-aminopropyltriethoxysilane (APTS, purity 99%), 4,4′-oxydianiline (ODA, purity 98%) and formaldehyde (37 wt %) were purchased from Acros Organics Co., Morris, NJ, USA. Anhydrous stabilized tetrahydrofuran (THF) was supplied by Lancaster Co., Morecambe, Lancashire, UK. Arcol polyol 1007 (polyether polyols 700) was purchased from Bayer Material Science Ltd., Kaohsiung, Taiwan. MPP (phase II, *n* > 1000) was purchased from San Jin Chemicals Corporation, Kaohsiung, Taiwan.

### 2.2. Preparation of Si-PU

**Scheme 1:**
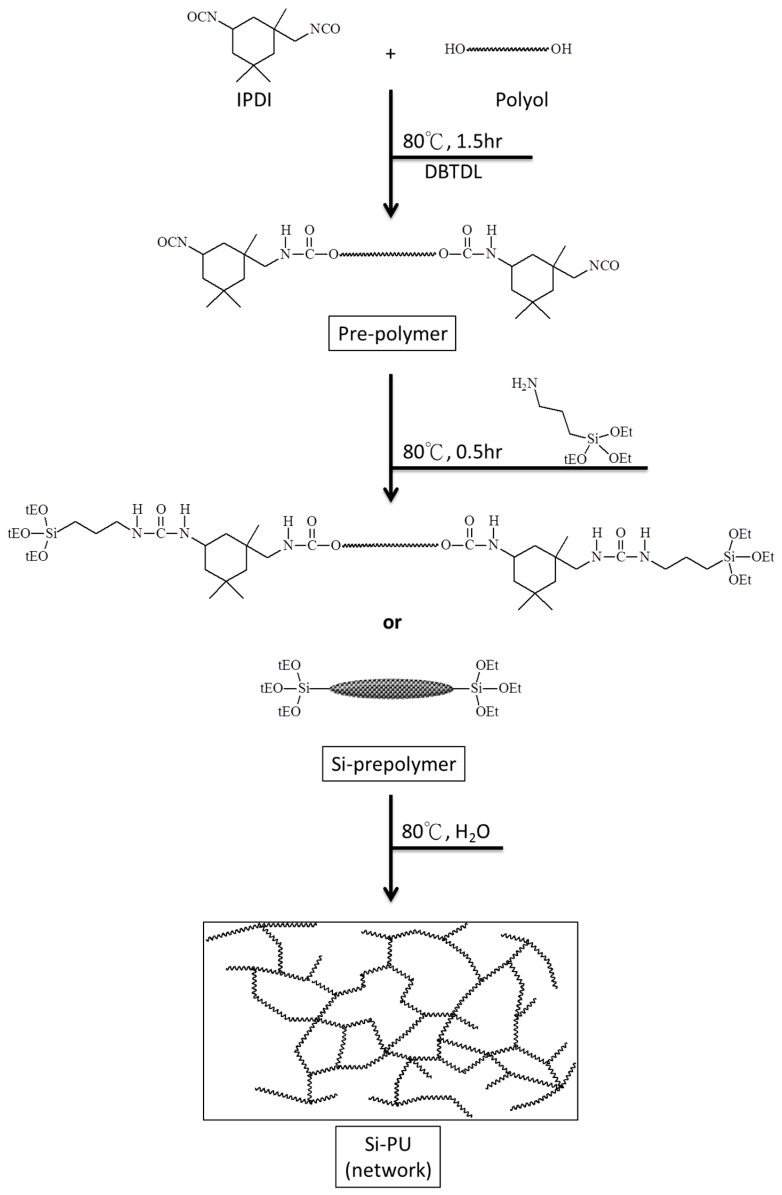
Preparation of Si-PU composites.

First, IPDI (12.6 g) and polyether polyol (20 g) were placed into a four-necked flask. The contents of the flask were mechanically stirred in a nitrogen atmosphere at 80 °C (external oil bath). Next, a metal catalyst (DBTDL, 1 g) was placed into the flask. The mixture was stirred for 1.5 h to generate a prepolymer and the viscosity reached 5000 cp. The temperature was lowered to 50 °C to avoiding the escapting of solvent. THF solvent (50 mL), APTS (12.6 g) were added to the mixture and it was stirred for 0.5 h, following which H_2_O (0.5 mL) was added. The temperature was increased to 70 °C and the viscosity increased due to increasing reaction extent. When the viscosity increased to 15,000 cp, the finished product (Si-PU) was removed from the reaction vessel, allowed to stand at room temperature for 6 h and then dried in an oven at 80 °C at a reduced pressure for 12 h. The reaction is shown in Scheme 1.

### 2.3. Preparation of OFMPP

**Scheme 2:**
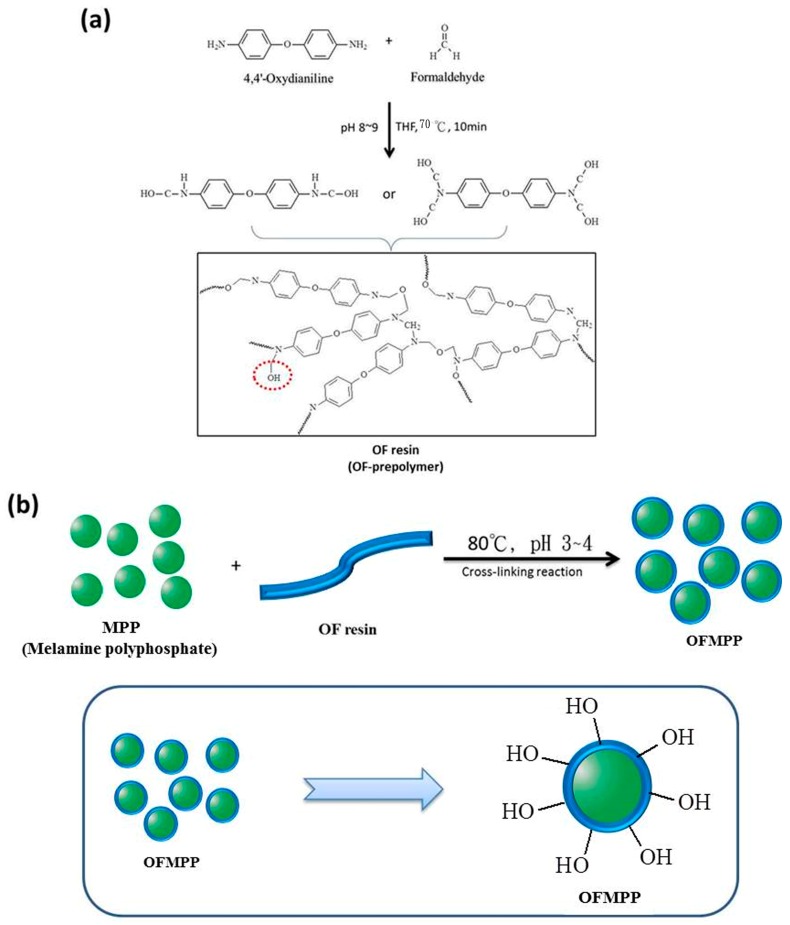
(**a**) Preparation of OF resin (**b**) Preparation of OFMPP.

First, 4,4′-oxydianiline (10 g) and formaldehyde (5.99 g) were placed into a reaction vessel and then THF (40 mL) was added. Next, aqueous ammonia solution was added to adjust the pH to 8–9. The temperature was increased to 60 °C for 10 min and the mixture became transparent. Next, MPP (40 g) and ethanol (500 mL) were placed in another reaction vessel, preheated and stirred for 0.5 h. The transparent prepolymer was added into the mixture. The pH of the mixture was adjusted to be between 3 and 4 through the addition of aqueous hydrochloric solution and it was stirred at 80 °C for 3 h. The solid product was collected by filtration at reduced pressure, washed with ethanol and dried in a convection oven for 12 h (Scheme 2).

### 2.4. Preparation of Si-PU/OFMPP Composites

**Scheme 3:**
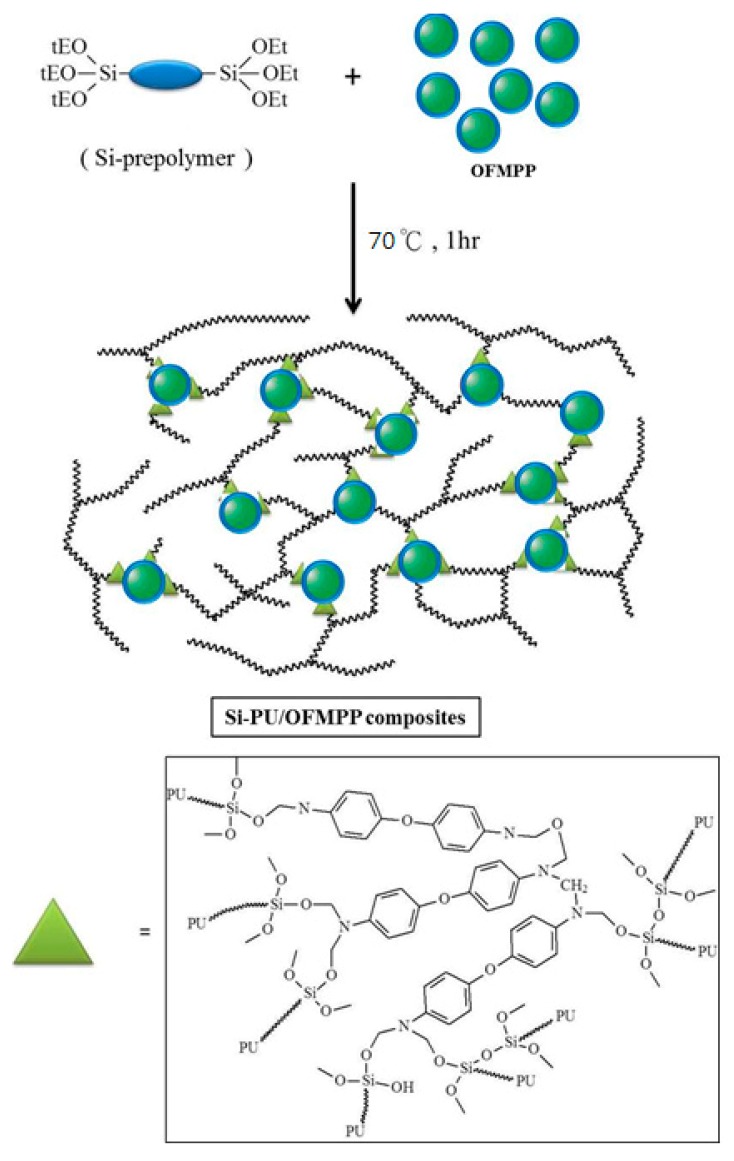
Preparation of Si-PU/OFMPP composites.

OFMPP flame retardant was added into the Si-prepolymer synthesized according to Scheme 3. Then, 0.5 mL of H_2_O was added and the temperature was increased to 70 °C. The finished product (Si-PU/OFAPP) was removed after the viscosity increased and placed into a mold. It was allowed to stand at room temperature for 6 h and then placed in an oven, following which it was dried for 12 h at 80 °C and pressure was reduced.

### 2.5. Measurements

^29^Si NMR was performed using a spectrometer (DSX-400WB, Bruker, Rheinstetten, Germany). The samples were treated at 180 °C for 2 h and then ground into fine powder. X-ray photoelectron spectra (XPS) were recorded using a PHI Quantera SXM/Auger with Al Ka excitation radiation (*h*_ν_ = 1486.6 eV). The pressure in the analyzer was maintained at approximately 6.7 × 10^−7^ Pa. XPS data were processed using a DS 300 data system. MPP or microencapsulated MPP samples (10 g) was placed into 100 mL distilled water at a different temperature and stirred at that temperature for 60 min. The suspension was then filtered and 50 mL of the filtrate was collected and dried to a constant weight at 105 °C. The morphology of the burnt surface of the composites was examined using a scanning electron microscope (SEM) (JEOL JSM 840A, Osaka, Japan). Thermal degradation of the composites was examined using a thermogravimetric analyzer (TGA) (Perkin Elmer TGA 7, PerkinElmer Co., Waltham, MA, USA) from room temperature to 800 °C at a heating rate of 10 °C/min in nitrogen atmosphere. The measurements were performed for 6–10 mg of samples. LOI testing was performed following the ASTM D 2836 Oxygen Index Method (Atlas Fire Science Products Co., Kent, WA, USA), by using a test specimen bar that was 15 cm long, 6.5 ± 0.5 mm wide and 3.0 ± 0.5 mm thick. The sample bars were suspended vertically and ignited using a Bunsen burner. The flame was removed and the timer was started. The concentration of oxygen increased when the flame on the specimen was extinguished before it had burned for 3 min or burned 5 cm away from the bar. The oxygen content was adjusted until the limiting concentration was determined.

The UL94 test method based on the UL Standard for Tests for Flammability of Plastic Materials for Parts in Devices and Appliances was used. Each test specimen bar was 15 cm long, 6.5 ± 0.5 mm wide and 3.0 ± 0.5 mm thick. The vertical burning test was performed inside a fume hood. The samples were held vertically with tongs at one end and burned from the free end. The samples were exposed to an ignition source for 10 s, following which they were allowed to burn above cotton wool until both the samples and cotton wool were extinguished. Observable parameters were recorded to assess the fire retardance. The UL 94 test classified the materials as V-0, V-1 and V-2 according to the time period required before self-extinction and the occurrence of flaming dripping after the ignition source was removed. Each specimen was supported such that its lower end was 10 mm above the Bunsen burner tube. V-0 is the most ambitious and desired classification. A blue 20-mm-high flame was applied to the center of the lower edge of the specimen for 10 s and then removed. If burning ceased within 30 s, the flame was reapplied for an additional 10 s. If the specimen dripped, particles were allowed to fall onto a layer of dry absorbent surgical cotton placed 300 mm below the specimen. The specimens were not allowed to burn with flames for more than 10 s after either application of the test flame. The specimens that did not drip flaming particles that would ignite the surgical cotton were classified as V-0 level. The specimens that did not burn with glowing or flames up to the holding clamp for more than 30 s after either application of the test flame and did not drip flaming particles that would ignite the surgical cotton were classified as V-1 level. The specimens that did not burn with flames for more than 30 s after either application of the test flame and dripped flaming particles that ignited the surgical cotton were classified as V-2 level. The specimens that burned with flames for more than 30 s after either application of the test flame were classified as Fail.

## 3. Results and Discussion

### 3.1. ^29^Si-NMR of Si-PU

The structure of the pure PU matrix was relatively weak and was easily destroyed when heated. After the structure was modified through the chemical reaction in Scheme 1, PU networks were formed that promoted thermal stability and flame retardance. During the structural formation of the Si-PU composite, a hydrolysis-condensation (sol-gel) reaction occurred. The degree of condensation was analyzed through solid-state ^29^Si-NMR to obtain the percentage of condensation density.

**Figure 1:**
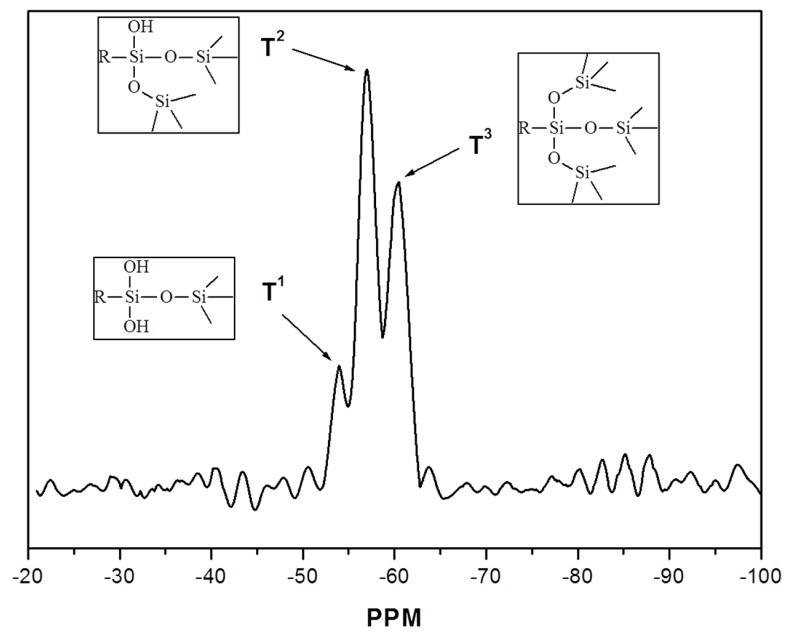
Solid-state ^29^Si-NMR spectra of Si-PU composites.

As shown in Figure 1, the *t*-distribution and size of silicon spectra were explored. *T*^1^ is a monosubstituted siloxane bond, *T*^2^ is a disubstituted siloxane bond and *T*^3^ is a trisubstituted siloxane bond, representing the number of bonds of the tri-alkoy group of APTS that underwent condensation. The figure clearly shows the area size of *t*-distribution at *T*^2^ > *T*^3^ > *T*^1^. After the peak separation, the area size data were input into Equation (1) [16] to obtain a condensation density of 76.33%, as shown in Table 1, indicating that Si–O–Si functional group has a favorable network structure [17]. Network structure promoted the thermal stability of the composites.

(1) $$D_{c}(\%)=\left[\frac{T^{1}+2T^{2}+3T^{3}}{3}+\frac{Q^{1}+2Q^{2}+3Q^{3}+4Q^{4}}{4}\right]\times 100$$

### 3.2. XPS of OFMPP

**Figure 2:**
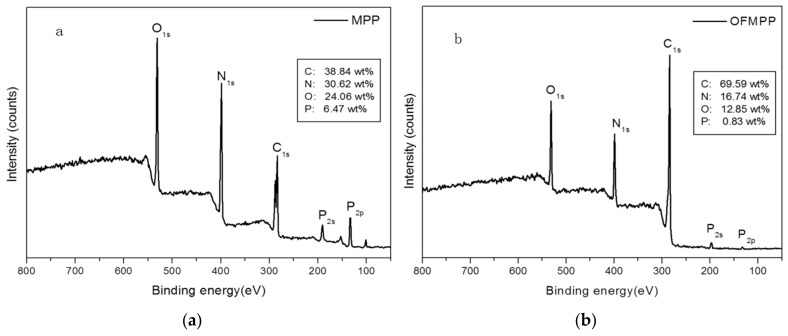
XPS spectra of: (**a**) MPP; and (**b**) OFMPP.

Figure 2 shows the peak chart produced by the XPS scan showing the microencapsulation of MPP. The P_2S_ and P_2P_ of MPP were 134 and 191 eV, respectively, clearly indicating the peaks. The intensities after microencapsulation were significantly reduced at P_2S_ and P_2P_. O_1s_ and N_1s_ peaks were also slightly reduced. This may be because the outer layer of OFMPP had a shell. By contrast, at C_1s_, the peaks of OFMPP were higher compared with those of MPP.

Thus, after microencapsulation, the outer surface of MPP has a layer of capsule shell, indicating that a new flame retardant, OFMPP, was successfully formed through the microencapsulation of MPP.

### 3.3. Water Solubility of OFMPP

**Figure 3:**
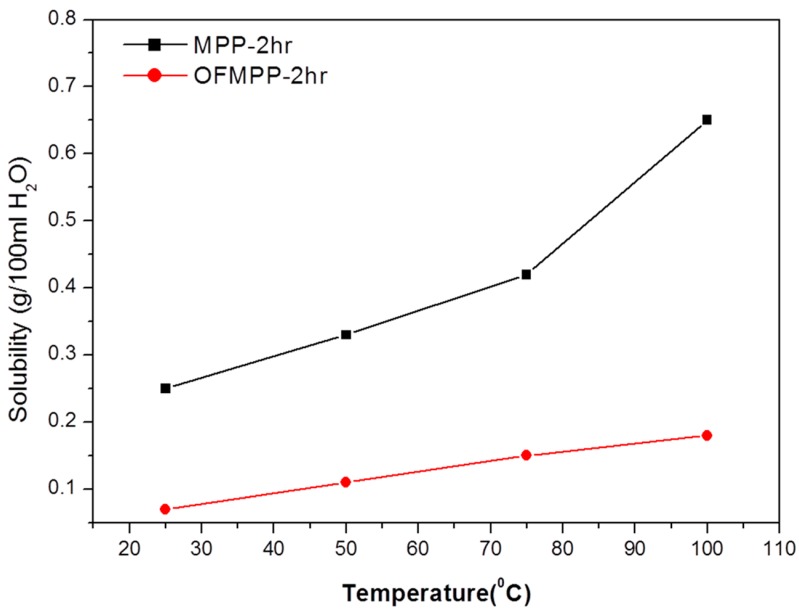
Water solubility of MPP and OFMPP.

The compatibility between fillers and polymer matrix was tested through water solubility. If the water solubility was poor, the fillers were hydrophobic. The compatibility between fillers and polymer matrix improved and the fillers were well dispersed in polymer matrix. Figure 3 and Table 2 present the weight change data of MPP and OFMPP after boiling in water solution. First, 1-g samples of each MPP and OFMPP were placed into four containers and 50 mL of DI water were added into each container. The temperature was increased to 25, 50, 75 and 100 °C (with 2 h of stirring), respectively. The samples were filtered and dried, following which the weight was measured to observe the change. If the weight remained constant, the compatibility with water was poor, indicating that the samples were hydrophobic [18,19,20,21,22,23,24]. If the weight was somewhat reduced, they were considered hydrophilic.

According to Figure 3 and Table 2, at room temperature, MPP and OFMPP lost 0.25 and 0.07 g/100 mL H_2_O, respectively, after 2 h of stirring. When the temperature was raised to 100 °C, they lost 0.65 and 0.18 g/100 mL H_2_O, respectively. This indicates that MPP after water boiling is highly compatible with water, i.e., it is hydrophilic. The weight of OFMPP did not change much after being boiled in water, indicating an increase in its hydrophobicity. The aforementioned data show that OFMPP does not have high water solubility after microencapsulation, indicating that it was not affected for the storage at room temperature by moisture to experience deliquescence and that the stability increased during storage. This implies that the OFMPP within the composites did not easily migrate in the humid environment and the flame retardance of the composites was maintained to a satisfactory degree. By contrast, the hydrophobic nature of OFMPP enhanced its compatibility with the Si-PU matrix.

### 3.4. TGA of OFMPP

**Figure 4:**
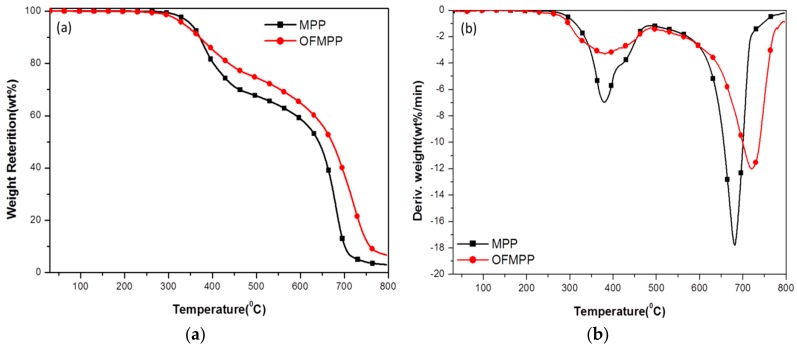
(**a**) TG; and (**b**) DTG curves of MPP and OFMPP.

Figure 4 shows the thermogravimetric (TG) and derivative thermogravimetric (DTG) graphs of MPP and OFMPP, which indicate that MPP and OFMPP have two stages of thermal degradation. For MPP, the first stage occurred from 270 to 450 °C, wherein it released NH_3_ and H_2_O. Simultaneously, melamine was converted into melam, melem, and melon, and MPP was gradually converted into pyrophosphate and polyphosphate. At the second stage at more than 500 °C, melam pyrophosphates or melam polyphosphates began to be changed from MPP and the final product was char residue containing P–N [3,5,23,24,25]. For OFMPP, the TG curve at less than 350 °C was very similar to that of MPP, but OFMPP had high thermal stability after 350 °C, with the degradation temperature decreasing. The DTG clearly shows that the degradation rates in the two stages significantly decreased. This may be because of the outer shell (OF) of OFMPP, where OF mainly comprised the benzene ring, thus increasing the thermal stability and delaying the temperature for thermal degradation. The char yields at 800 °C for MPP and OFMPP were 2.9 and 6.7 wt %, respectively, where OFMPP improved the char formation by 3.8 wt %.

The curves of TG and DTG show that the conversion of MPP into OFMPP through microencapsulation indicated high thermal stability, implying that OFMPP can have a strong effect.

### 3.5. LOI and UL-94 of Si-PU/OFMPP Composites

**Figure 5:**
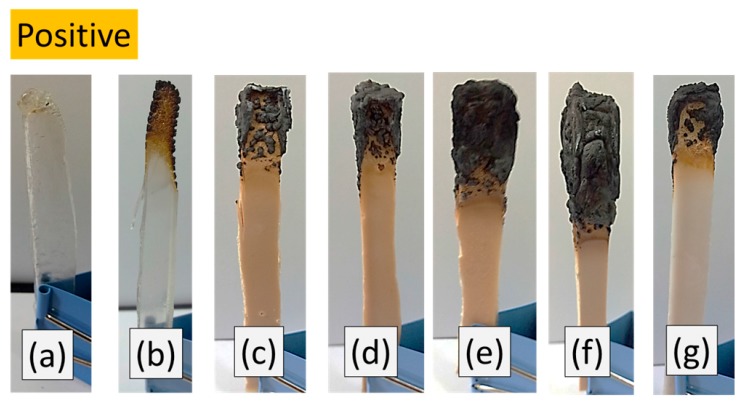
Residues after burning for 1 min: (**a**) pure PU; (**b**) Si-PU; (**c**) Si-PU/OFMPP 10%; (**d**) Si-PU/OFMPP 20%; (**e**) Si-PU/OFMPP 30%; (**f**) Si-PU/OFMPP 40% and (**g**) Si-PU/MPP 30%.

**Figure 6:**
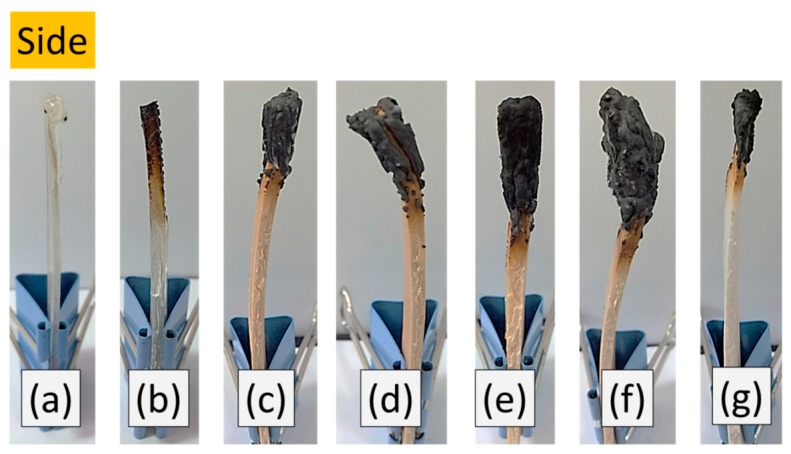
Residues after burning for 1 min: (**a**) pure PU; (**b**) Si-PU; (**c**) Si-PU/OFMPP 10%; (**d**) Si-PU/OFMPP 20%; (**e**) Si-PU/OFMPP 30%; (**f**) Si-PU/OFMPP 40% and (**g**) Si-PU/MPP 30%.

Figure 5 and Table 3 present the experimental results for pristine PU, Si-PU, Si-PU/MPP 30%, and Si-PU/OFMPP 10–40% after LOI and UL-94 tests. Figure 5 and Figure 6 show the actual appearance after the composites were burned.

Figure 5 and Table 3 show that the LOI value of pristine PU was 17%, which was improved to 18% for modified Si-PU. For the composites with added OFMPP at 10%, 20%, 30% and 40%, the LOI values were 19%, 25%, 32% and 38%, respectively; the LOI values were greatly improved after OFMPP was added. In terms of UL-94, pristine PU could not pass the flame retardant test. When the OFMPP concentration was 20 wt %, the UL-94 test result was still Fail. The specimens burned with flaming combustion for more than 30 s after either application of the test flame. However, at 30% concentration, the results of UL-94 showed significant changes and improvement to the highest V-0 grade. A comparison of the flame retardance between Si-PU/MPP 30% and Si-PU/OFMPP 30% showed that the LOI and UL-94 for Si-PU/MPP 30% were 27% and Fail, respectively, and 32% and V-0 for Si-PU/OFMPP 30%. The LOI of Si-PU/OFMPP 30% was 5% higher than that of Si-PU/MPP 30%; UL-94 was the most obvious, with Fail improved to V-0. This indicates that OFMPP offers outstanding performance compared with MPP in terms of flame retardance.

In Table 3, the LOI value for Si-PU/MPP 30% before hot water treatment was 27% and was reduced to 24% after hot water treatment (75 °C for 24 h). For Si-PU/OFMPP 30%, the LOI value was reduced to 30% from the original 32%. The LOI change for Si-PU/MPP 30% was more significant than that for Si-PU/OFMPP 30%. By contrast, in terms of the change for UL-94, the grade for Si-PU/OFMPP 30% changed from V-0 to V-1.

As for the appearance change after burning, the front view in Figure 5 shows that the top of pristine PU after burning was in a candle-like molten state without any char-forming effect, whereas Si-PU had char residue after burning. The composite with OFMPP showed that as the concentration was increased, the char layer became more obvious and the intumescent volume increased. By contrast, the char-forming effect for the composites with added MPP 30% was different from that of OFMPP 30%. Si-PU/MPP 30% showed partial char, whereas the char for Si-PU/OFMPP 30% was more compact and wrapped around the entire polymer substrate.

The side view in Figure 6 shows the intumescent effect during the burning process. Both pristine PU and Si-PU showed no intumescent flame-retardant behavior, whereas the intumescent effect became more obvious for Si-PU/OFMPP 10–40% as the concentration increased, indicating higher flame retardance. Irrespective of the size or width of the intumescent area, Si-PU/OFMPP 30% had higher performance than Si-PU/MPP 30%. To summarize the results from LOI and UL-94, the comparison showed that OFMPP, a product after microencapsulation, was more effective than MPP in terms of flame retardance, implying that microcapsule formation improved the barrier effect. Furthermore, the composites with added OFMPP had satisfactory flame retardance, whereas those with added MPP had lower performance. Therefore, in terms of hot water treatment, OFMPP after microencapsulation offered higher water resistance. When it was added into the polymer matrix, the flame retardance did not change much, meaning that OFMPP was apparently more effective than MPP and that the microencapsulation technology is flame retardant to a certain extent.

### 3.6. SEM of Si-PU/OFMPP Composites after Burning 

**Figure 7:**
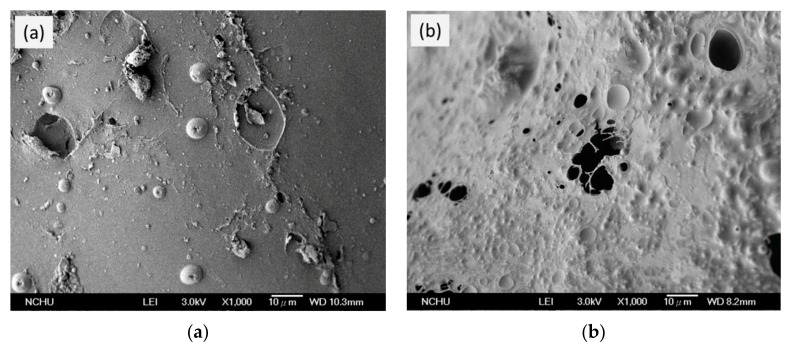

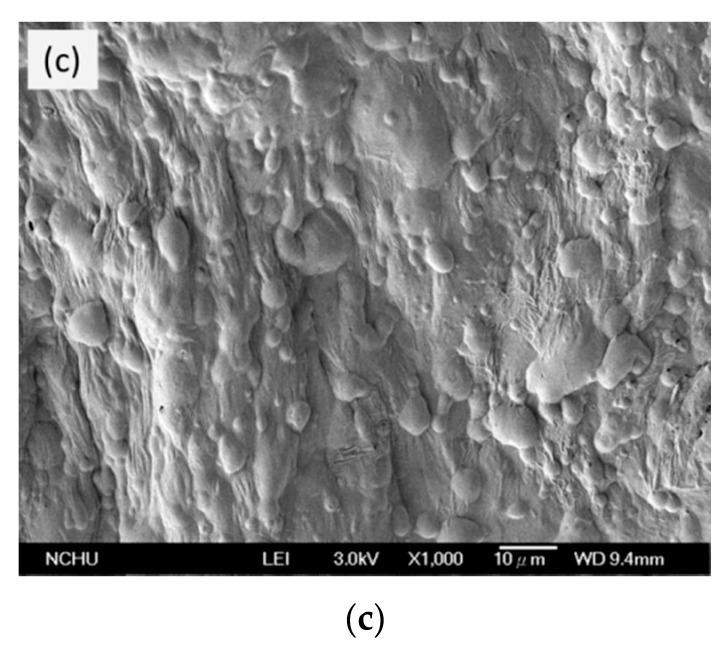
SEM images of char residues after burning: (**a**) Si-PU; (**b**) Si-PU/MPP 30% and (**c**) Si-PU/OFMPP 30%.

Figure 7 shows SEM images of the morphology of Si-PU, Si-PU/MPP 30% and Si-PU/OFMPP 30% after burning. Figure 7a shows intumescent pores and hollow voids on the surface of the Si-PU matrix after burning. The intumescent pores were formed by the char layer that was pushed when nonflammable gas from the polymer was released. The voids were originally the intumescent pores that burst after the char layer failed to withstand the strong release of the nonflammable gas. The formation of voids introduces oxygen into the polymer substrate, further burning the matrix so that an effective barrier cannot form to protect the polymer matrix. Figure 7b also shows voids in the char layer after MPP is added. This may be because MPP causes the release of nonflammable gases, for example, water vapor and NH_3_. However, there are no pores, indicating that MPP’s nitrogen ring in melamine sufficiently complements the compactness of the char layer, although the layer thickness is inadequate. Figure 7c shows that with OFMPP, the char density on the surface is sufficient and there are intumescent layers that do not burst. This indicates that the benzene ring structure on the outer shell of OFMPP has a sufficiently thick char layer to form a protective layer during burning [26,27,28,29,30]. This can support nonflammable air and the char layer stops the release of air while generating an intumescent flame-retardant layer.

Thus, it can be concluded that Si-PU is a poor flame retardant, flame retardance of the composite is still inadequate only after MPP is added and the composite with MPP after microencapsulation can achieve compactness and sufficient barrier thickness. This means that the char layer of OFMPP is significantly more favorable than that of MPP. Thus, OFMPP can effectively enhance flame retardance and form an excellent thermal barrier.

## 4. Conclusions

The new generation of microencapsulation flame retardant developed in this study involved the OF resin wrapping around the surface of the MPP to form an OFMPP flame retardant through the characterization of XPS, ^29^Si NMR and water solubility. The data from the tests showed that adding Si-PU/OFMPP 40% to polymer matrix could make the char yields reach approximately 26.9 wt % and flame retardance grades LOI-38% and UL-94 V-0, indicating high performance. The test curves of TGA and SEM also proved that OFMPP produced protective mechanisms at different temperatures, further improving the thermal stability and char yield. When MPP before and after microencapsulation are compared, the treatment clearly demonstrates high flame retardance and thermal stability. The new microencapsulated flame retardant successfully improved the shortcomings of flammable PU and could be developed for use in various applications.

## Figures and Tables

**Table 1:** Area of the t-distribution.

Sample No.	Area (%)			Degree of condensation (%)
	*T*^1^	*T*^2^	*T*^3^	
Si-PU composites	8	55	37	76.33

**Table 2:** Water solubility data of MPP and OFMPP.

Sample code	25 °C (g/100 mL H_2_O)	50 °C (g/100 mL H_2_O)	75 °C (g/100 mL H_2_O)	100 °C (g/100 mL H_2_O)
MPP	0.25	0.33	0.42	0.65
OFMPP	0.07	0.11	0.15	0.18

**Table 3:** Flame-retardant properties of pure PU, Si-PU, Si-PU/MPP 30% and Si-PU/OFMPP 10–40% composites.

Sample No.	LOI (%)		UL-94			
	Before soakage	After soakage	Before soakage		After soakage	
			Ranking	Dripping	Ranking	Dripping
Pristine PU	17	17	Fail	YES	Fail	YES
Si-PU	18	18	Fail	NO	Fail	NO
Si-PU/OFMPP 10%	19	19	Fail	NO	Fail	NO
Si-PU/OFMPP 20%	25	24	Fail	NO	Fail	NO
Si-PU/OFMPP 30%	32	30	V-0	NO	V-1	NO
Si-PU/OFMPP 40%	38	36	V-0	NO	V-0	NO
Si-PU/MPP 30%	27	24	Fail	NO	Fail	NO
